# Effectiveness and safety of tripterygium wilfordii poly-glycosides on glomerulonephritis: a systematic review and meta-analysis

**DOI:** 10.3389/fphar.2024.1339153

**Published:** 2024-05-22

**Authors:** Xiaolin Yan, Juan Shi, Yingying Zhang, Juan Liu, Xiaoqing Lin, Chungang Yu, Xiao Li

**Affiliations:** ^1^ Department of Clinical Pharmacy, The First Affiliated Hospital of Shandong First Medical University and Shandong Provincial Qianfoshan Hospital, Shandong Engineering and Technology Research Center for Pediatric Drug Development, Shandong Medicine and Health Key Laboratory of Clinical Pharmacy, Jinan, China; ^2^ Department of Pharmacy, The First People’s Hospital of Jinan, Jinan, China

**Keywords:** tripterygium wilfordii poly-glycosides, membranous nephropathy, diabetic kidney disease, henoch-schonlein purpura nephritis, safety

## Abstract

Treatment of glomerulonephritis presents several challenges, including limited therapeutic options, high costs, and potential adverse reactions. As a recognized Chinese patent medicine, *Tripterygium wilfordii* poly-glycosides (TWP) have shown promising benefits in managing autoimmune diseases. To evaluate clinical effectiveness and safety of TWP in treating glomerulonephritis, we systematically searched PubMed, Cochrane Library, Web of Science, and Embase databases for controlled studies published up to 12 July 2023. We employed weighted mean difference and relative risk to analyze continuous and dichotomous outcomes. This meta-analysis included 16 studies that included primary membranous nephropathy (PMN), type 2 diabetic kidney disease (DKD), and Henoch-Schönlein purpura nephritis (HSPN). Analysis revealed that additional TWP administration improved patients’ outcomes and total remission rates, reduced 24-h urine protein (24hUP) and decreased relapse events. The pooled results demonstrated the non-inferiority of TWP to glucocorticoids in achieving total remission, reducing 24hUP, and converting the phospholipase A2 receptor (PLA2R) status to negative. For DKD patients, TWP effectively reduced 24hUP levels, although it did not significantly improve the estimated glomerular filtration rate (eGFR). Compared to valsartan, TWP showed comparable improvements in 24hUP and eGFR levels. In severe cases of HSPN in children, significant clinical remission and a reduction in 24hUP levels were observed with the addition of TWP treatment. TWP did not significantly increase the incidence of adverse reactions. Therefore, TWP could offer therapeutic benefits to patients with PMN, DKD, and severe HSPN, with a minimal increase in the risk of side effects.

## 1 Introduction

Glomerulonephritis is one of the leading causes of chronic kidney disease (CKD), affecting approximately 132.3 million individuals in China (Collaboration, 2020). Treatment options are limited and include renin-angiotensin system (RAS) blockers, glucocorticoids, cyclophosphamide, calcineurin inhibitors, and rituximab. These are selected based on clinical symptoms, laboratory tests, and renal biopsy results (Group, 2021). However, the adverse events and high costs associated with these medications often result in poor patient compliance. For example, long-term glucocorticoid use may cause obesity and hyperglycemia. Cyclophosphamide therapy carries a high risk of marrow suppression, hemorrhagic cystitis, and malignancy. Cyclosporin A is limited by its potential to cause secondary fibrosis and tubular atrophy in the renal interstitium. Patients treated with tacrolimus often experience relapses after discontinuation of the drug (Group, 2021). The high rituximab cost makes it unaffordable to many, particularly in developing countries. Furthermore, low effectiveness and dependence on steroids can lead to decreased hope and compliance among patients. Therefore, there is a critical need for new therapeutic agents.

Despite an incomplete understanding of the pathogenesis of glomerulonephritis, emerging evidence suggests that inflammatory cell infiltration into the glomerular capillaries and activation of resident glomerular cells are typically involved in the development of common glomerulonephritides, ultimately leading to damage to the glomerular filtration barrier (GFB) (Couser, 2012). *Tripterygium wilfordii* polyglycosides (TWP) are a mixture of natural active ingredients extracted from the root xylem of the *T. wilfordii* herb. The efficacy of TWP in reducing proteinuria in patients with glomerulonephritis was first described in 1977. In addition to being cost-effective, TWP exhibits potent anti-inflammatory, immunomodulatory, and podocyte-protective properties (Liu et al., 2021; Wang et al., 2021; Shi et al., 2022). It has been used to treat autoimmune and inflammatory diseases, such as rheumatoid arthritis and nephrotic syndrome (Wang et al., 2017). However, the extent of the effectiveness of TWP in reducing proteinuria and preserving renal function, particularly compared to other medications, remains controversial. Moreover, concerns about multi-organ toxicity associated with TWP persist. This meta-analysis and systematic review compile the current evidence on the effectiveness and safety of TWP in treating common glomerular disorders from controlled studies.

## 2 Materials and methods

This meta-analysis followed the Preferred Reporting Items for Systematic Reviews and Meta-analyses (PRISMA) reporting guidelines (Moher et al., 2009). The study protocol was registered in PROSPERO (Registration number: CRD42023461828).

### 2.1 Data sources and search strategy

Using a combination of subject headings and free terms, we conducted a comprehensive literature search for studies on common glomerulonephritides treated with TWP. The search was performed using the PubMed, Cochrane Library, Web of Science, and Embase databases, covering all publications from their inception through 12 July 2023. Detailed search strategies are provided in the Supplementary Material. The reference lists of included studies and relevant review articles were examined to identify further pertinent studies. The literature retrieval was carried out independently by two investigators.

### 2.2 Inclusion criteria

The inclusion criteria were established based on PICOS principles. A study was eligible if it simultaneously met the following criteria: (1) Participants: Human subjects diagnosed with primary or secondary glomerulonephritis; (2) Interventions and Comparisons: The TWP group received basic therapeutics plus TWP, while the control group received basic therapeutics alone or combined with another drug. Other measures remained consistent across both groups; (3) Outcome Measurements: Studies were included if they analyzed at least one of the following: clinical remission rate, relapse, 24-h urine protein (24hUP), estimated glomerular filtration rate (eGFR), serum creatinine (SCr), blood albumin (Alb), and adverse drug reactions (ADRs). Total clinical remission was defined as a reduction of 24hUP to less than 3.5 g, or at least a 50% reduction from the peak value. Relapse was defined as an increase in 24hUP to more than 3.5 g or more than 50% from the lowest level during the treatment period; (4) Study Design: Only randomized controlled trials (RCTs), cohort studies or other controlled studies published in English were considered. If studies had missing or insufficient data, attempts were made to contact the authors by email to request the necessary information. Studies were excluded if this failed. In the cases of multiple publications of the same trial, only the publication with the most comprehensive information was selected. Several researchers independently screened the search results and cross-checked eligible studies. Any disagreements were resolved through consultation.

### 2.3 Exclusion criteria

A study was excluded if it met any one of the following criteria: (1) The type of study was a trial protocol, conference abstract, or case report; (2) Participants were treated with *T. wilfordii* herb extracts or dispensing granules; (3) Data of interest were not obtainable; (4) Baseline data between the TWP group and the control group were incomparable; and (5) Participants were diagnosed only with chronic nephritis or nephrotic syndrome, and not with a specific type of glomerulonephritis.

### 2.4 Data collection and quality assessment

The following data were extracted from the included studies: first author, publication year, study design, age range of participants, specific type of glomerulonephritis, baseline urinary protein level, sample size, interventions, TWP dose, observation duration, and outcomes. Quality assessment was conducted for cohort studies using the Newcastle-Ottawa Scale (NOS) (Oremus et al., 2012). The studies were categorized as high-quality (score of 7–9), medium-quality (score of 4–6), or low-quality (score of 0–3) based on their NOS scores. For RCTs, the modified Jadad Scale was used for quality evaluation (Oremus et al., 2012), classifying studies as high-quality (score of 4–8) or low-quality (score of 0–3). Two researchers independently assessed the quality of each study, and any discrepancies were resolved through discussion. If necessary, decisions were deferred to the corresponding author for final resolution.

### 2.5 Statistical analysis

Statistical analyses were conducted using Stata software, version 14.0. Heterogeneity among the pooled studies was evaluated using the Q test and quantified with the I^2^ statistic. Homogeneity was assumed if *p* ≥ 0.1 or I^2^ ≤ 50%, and the fixed-effect model was applied to compute the pooled results. A random-effects model was used in the cases of significant heterogeneity (*p* < 0.1 or I^2^ > 50%). For dichotomous outcomes, the relative risk (RR) with a 95% confidence interval (CI) was calculated to determine the effect size using the Mantel-Haenszel method for the fixed-effects model and the DerSimonian-Laird method for the random-effects model. The weighted mean difference (WMD) was used as the effect size indicator for continuous variables. The WMD and its 95% CI were estimated using the inverse variance method in the fixed-effects model and the DerSimonian-Laird method in the random-effects model. Statistical significance was determined by a *p*-value less than 0.05. Furthermore, if data permitted, subgroup and sensitivity analyses were conducted to explore potential sources of heterogeneity.

## 3 Results

### 3.1 Search results

**FIGURE 1:**
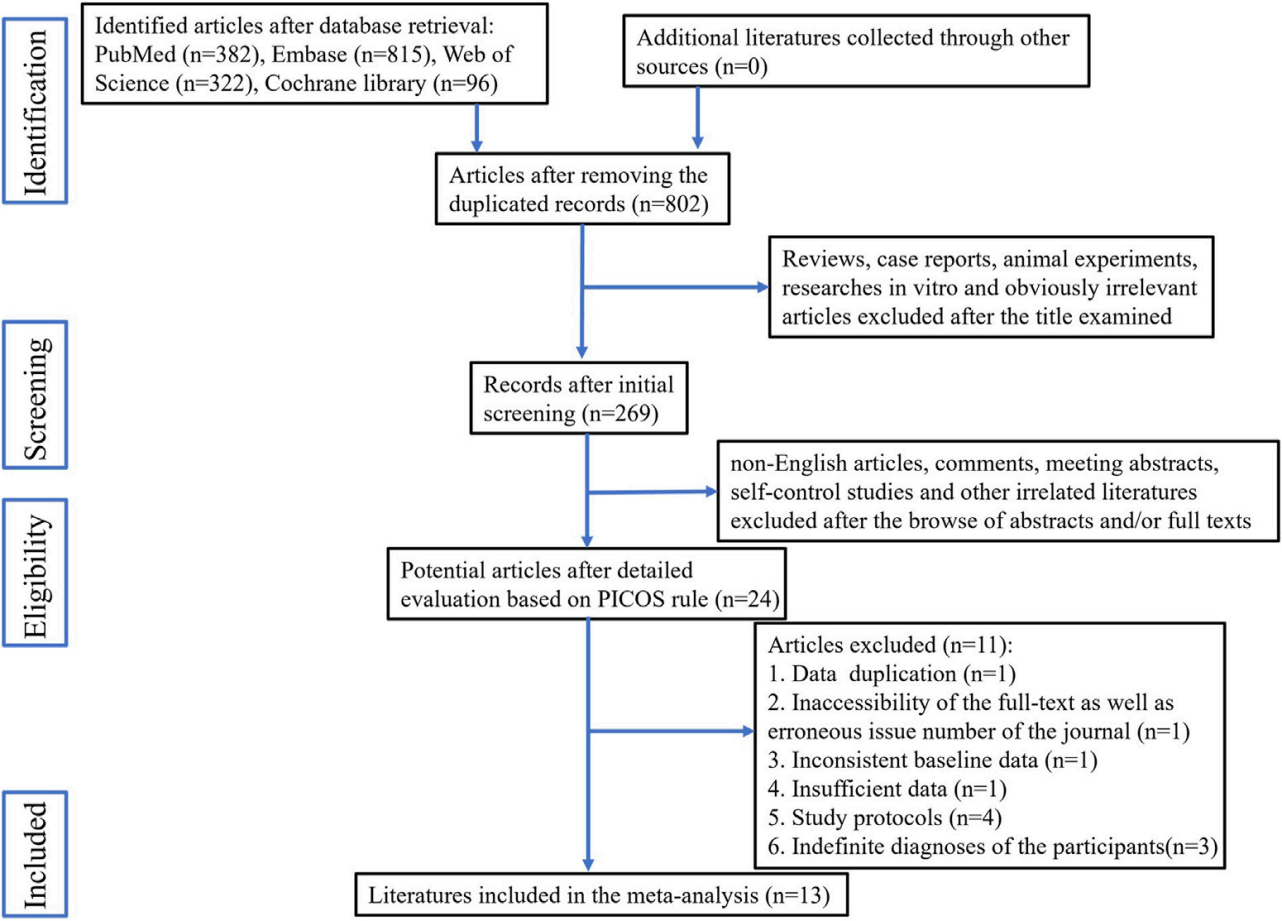
Flow chart of the publication identification and selection.

The study selection process is illustrated in Figure 1. A total of 14 studies met the eligibility criteria. Attempts were made to contact the authors of three articles for additional data by email; however, no responses were received. Consequently, one article was excluded due to the unavailability of the necessary data, while the other two were retained because they provided data for some of the required indicators. Ultimately, 13 articles were included in this meta-analysis (Deng et al., 2010; Deng et al., 2012; Ge et al., 2013; Wu et al., 2013; Liu et al., 2015; Shang et al., 2018; Wang, 2018; Xiong et al., 2020; Gao et al., 2021; Guo et al., 2021; Jin et al., 2021; Zhang et al., 2021; Wang et al., 2022). Of these articles, ten featured two parallel groups, while the remaining three included three parallel groups. In total, the 13 articles included 16 individual studies.

### 3.2 Description of the included studies

The main characteristics of the 16 studies are summarized in Table 1. These studies included three types of glomerulonephritides: primary membranous nephropathy (PMN), type 2 diabetic kidney disease (DKD), and Henoch-Schönlein purpura nephritis (HSPN). All trials were conducted in China and provided detailed descriptions of patient enrollment and withdrawal. Fifteen of the studies were cohort studies or RCTs. The remaining study was a prospective controlled open-label study, and quality was assessed using NOS (Wu et al., 2013), with specific quality score points provided in the Supplementary Material. Quality scores for prospective controlled open-label and cohort studies ranged from six to 8, while those for RCTs ranged from 5 to 6. Consequently, all included studies were of moderate-to-high quality.

**TABLE 1:** Characteristics of the included studies in the final analysis.

Study	Year	Study design	Age range	Type of glomerulonephritis	Urinary protein	Intervention of TWP group besides basic treatment	TWP dosage	Case number	Intervention of control group besides basic treatment	Case number	Duration	Quality score	Relevant outcomes
Shanshan Liu	2015	Prospective cohort	≥18	PMN	≥3.5 g/24 h	TWP plus prednisone	20 mg, tid	23	Tacrolimus plus prednisone	30	36 weeks	7	Clinical remission, 24hUP, Alb, number of relapsed cases, ADRs
Shunlai Shang	2018	Retrospective cohort	18–70	PMN	>3.5 g/24 h	TWP plus tacrolimus	20 mg, tid	21	Tacrolimus	33	10 months at least	8	Clinical remission, 24hUP, number of relapsed cases, negative conversion number of PLA2R, ADRs
Shunlai Shang	2018	Retrospective cohort	18–70	PMN	>3.5 g/24 h	TWP plus tacrolimus	20 mg, tid	21	Methylprednisolone plus tacrolimus	24	10 months at least	8	Clinical remission, 24hUP, number of relapsed cases, negative conversion number of PLA2R, ADRs
Ying Gao	2021	Retrospective cohort	18–70	PMN	>3.5 g/24 h	TWP plus tacrolimus/cyclosporin	average 20 mg, tid	33	Prednisone/Methylprednisolone plus tacrolimus/cyclosporin	31	12 months at least	7	Clinical remission, 24hUP, Alb, SCr, negative conversion number of PLA2R, ADRs
Yuanyuan Guo	2021	Retrospective cohort	18–65	PMN	1–3.5 g/24 h	TWP plus ARB	20 mg, tid	35	ARB	20	9 months at least	8	Clinical remission, 24hUP, eGFR, Alb, number of relapsed cases, ADRs
Yongchun Ge	2013	RCT	30–65	Type 2 diabetic kidney disease	≥2.5 g/24 h	TWP	average 30 mg, tid	29	Valsartan	26	6 months	5	24hUP, Alb, eGFR, ADRs
Wei Wang	2018	RCT	49–66	Type 2 diabetic kidney disease	>1 g/24 h	TWP	30 mg/d	20	None	20	6 months	6	24hUP, Alb, SCr, TNF-α, ADRs
Wei Wang	2018	RCT	49–65	Type 2 diabetic kidney disease	>1 g/24 h	TWP	60 mg/d	20	None	20	6 months	6	24hUP, Alb, SCr, TNF-α, ADRs
Chang Xiong	2019	RCT	30–70	Type 2 diabetic kidney disease	>3 g/24 h	TWP plus valsartan	60 mg/d	59	Valsartan	59	24 weeks	6	24hUP, Alb, eGFR, SCr, ADRs
Youyun Wang	2022	Retrospective cohort	30–70	Type 2 diabetic kidney disease	>3 g/24 h	TWP plus valsartan	20 mg, tid	158	Valsartan	127	24 weeks	8	24hUP, Alb, eGFR, SCr, ADRs
Youyun Wang	2022	Retrospective cohort	30–70	Type 2 diabetic kidney disease	>3 g/24 h	TWP	average 30 mg, tid	165	Valsartan	127	24 weeks	8	24hUP, Alb, eGFR, SCr, ADRs
Fang Deng	2010	Retrospective cohort	2–14	Children with HSPN	≥2 g/L	TWP plus methylprednisolone	1 mg/kg/day	24	methylprednisolone	14	3–6 months	6	Short-term clinical outcome, remission time of proteinuria, ADRs
Fang Deng	2012	Retrospective cohort	Pediatric patients	Children with HSPN	>40 mg/m^2^/h	TWP plus methylprednisolone	1 mg/kg/day	24	methylprednisolone	14	3–6 months	7	Short-term clinical outcome, remission time of proteinuria, ADRs
Li Wu	2013	prospective controlled open-label study	Pediatric patients	Children with HSPN	>40 mg/m^2^/h	TWP plus prednisone	1 mg/kg/day	42	prednisone	14	3–6 months	7	Short-term clinical outcome, ADRs
Yan Jin	2021	Retrospective cohort	3–15	Children with HSPN	unknown	TWP plus low molecular weight heparin	1.5 mg/kg/day	32	low molecular weight heparin	32	12 weeks	6	Short-term clinical outcome, 24hUP, urine red blood cell count, incidence of kidney damage
Huiwu Zhang	2021	RCT	4–18	Children with HSPN	≥50 mg/kg	TWP plus tacrolimus	almost 1.2 mg/kg/day	85	tacrolimus	87	6 months	6	24hUP, SCr, urine red blood cell count, ADRs

PLA2R: phospholipase A2 receptor; ARB: angiotensin receptor blocker; TNF-α: tumor necrosis factor α.

### 3.3 Effectiveness of TWP on membranous nephropathy

Four retrospective cohort studies and one prospective cohort study assessed the therapeutic outcomes of TWP in PMN (Liu et al., 2015; Shang et al., 2018; Gao et al., 2021; Guo et al., 2021). All participants received TWP at a dose of 60 mg per day. The studies were categorized into three groups based on the interventions: Group 1: TWP plus routine medicines *versus* routine medicines alone; Group 2: TWP plus routine medicines *versus* glucocorticoids plus routine medicines; Group 3: TWP plus routine medicines *versus* tacrolimus plus routine medicines.

**FIGURE 2:**
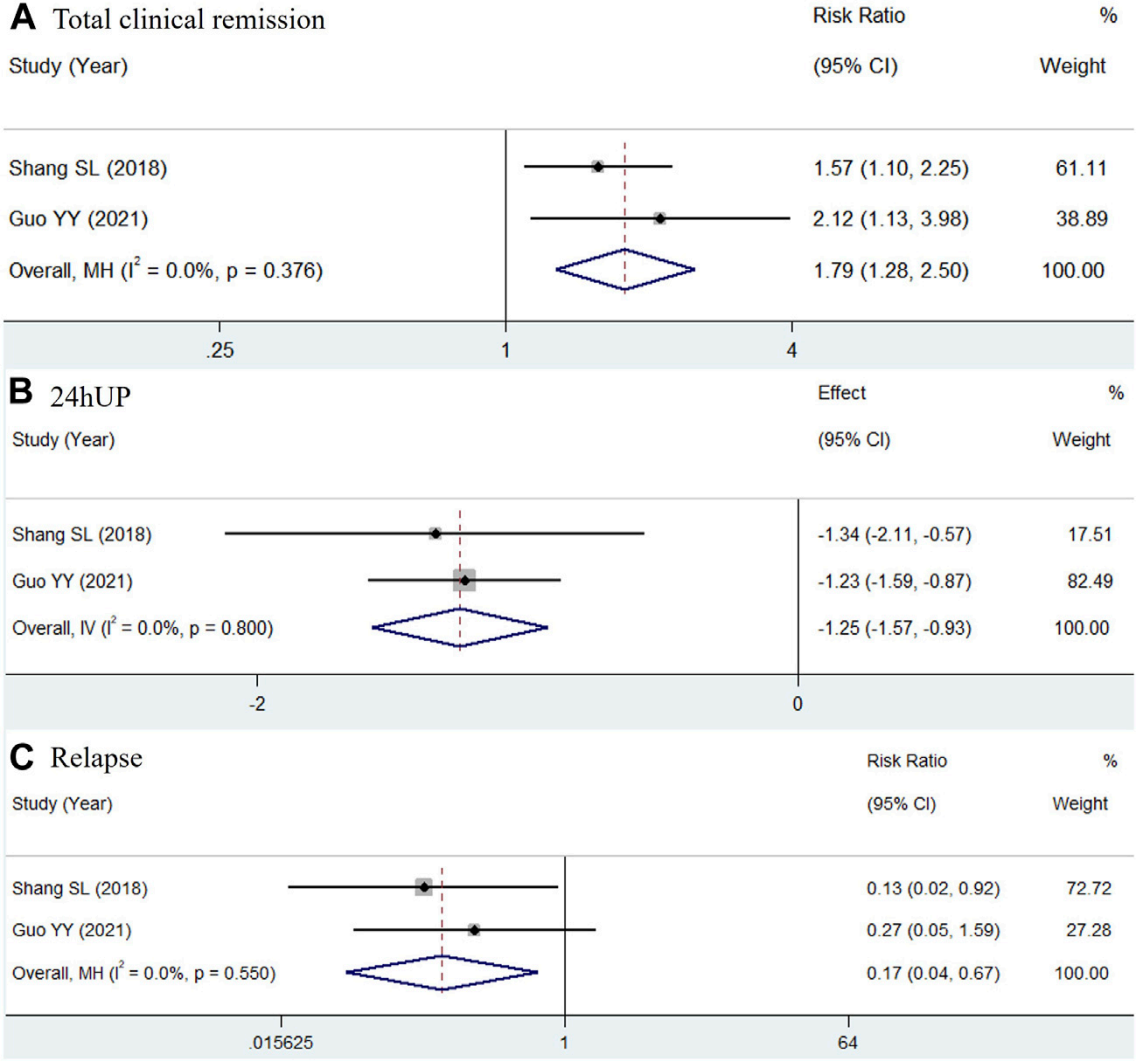
Forest plots of total clinical remission **(A)**, 24hUP **(B)** and relapse **(C)** of additional TWP on PMN. The efficacy of TWP + routine medicines was compared with that of routine medicines.

For Group 1, two studies with a minimum therapy duration of 9 months were included in a meta-analysis (Shang et al., 2018; Guo et al., 2021). The pooled results for total clinical remission, 24hUP, and relapse were analyzed and are shown in Figure 2. Due to the insignificant heterogeneity, a fixed-effect model was used. The findings showed significant benefits of adding TWP to routine medicines, with the pooled results indicating an increase in total clinical remission (RR = 1.79, 95% CI: 1.28–2.50, I^2^ = 0.0%), a decrease in 24hUP (WMD = −1.25, 95% CI: −1.57 to −0.93, I^2^ = 0.0%), and reduced relapse rates (RR = 0.17, 95% CI: 0.04–0.67, I^2^ = 0.0%).

**FIGURE 3:**
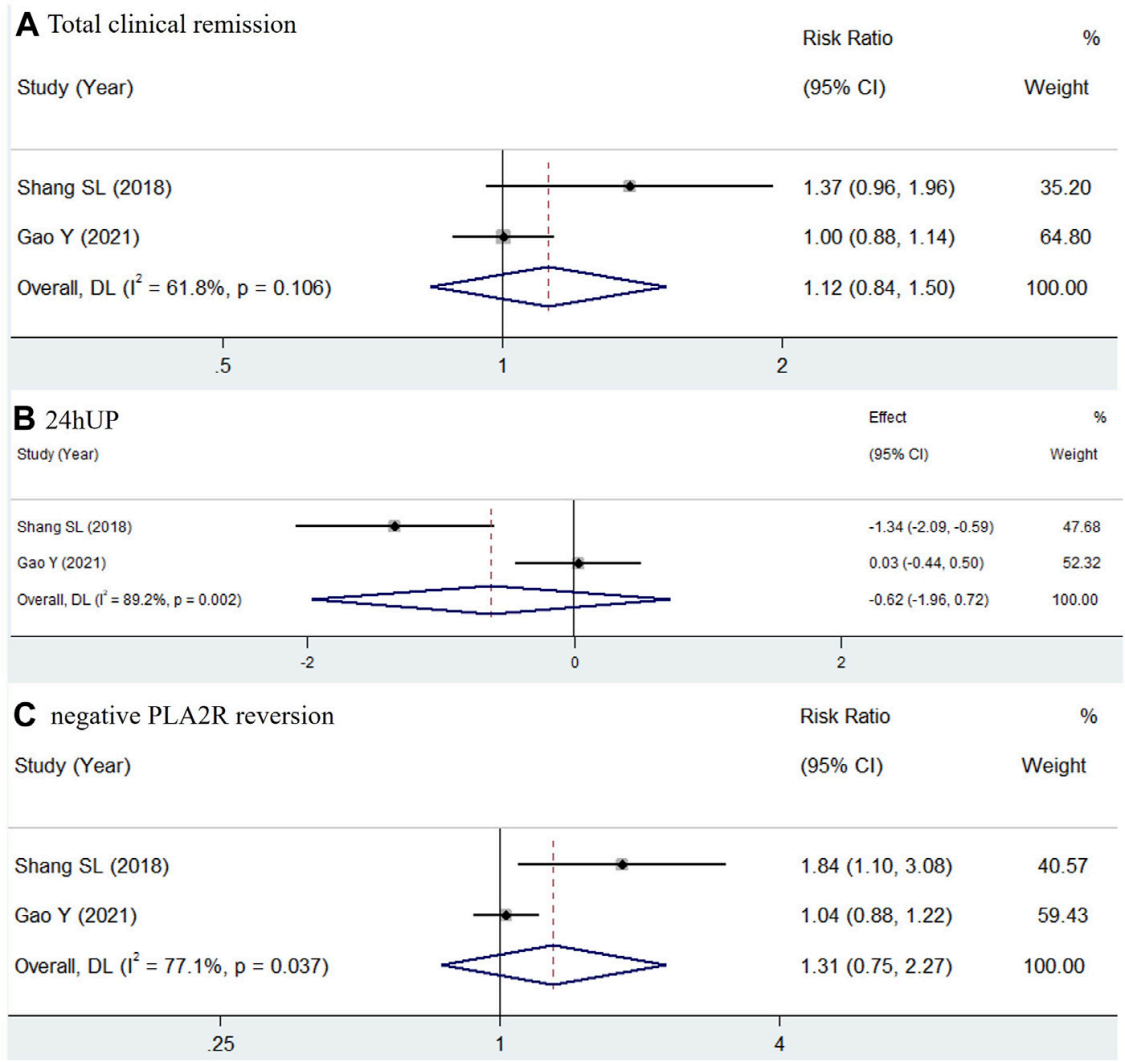
Forest plots of total clinical remission **(A)**, 24hUP **(B)** and negative PLA2R reversion **(C)** of TWP in comparison of glucocorticoid on PMN. The trial and control group received TWP + routine medicines and glucocorticoid + routine medicines, respectively.

For Group 2, two studies with a minimum therapy duration of 10 months were included in another meta-analysis (Shang et al., 2018; Gao et al., 2021). The results, shown in Figure 3, revealed significant heterogeneity; thus, a random-effects model was used. The outcomes did not demonstrate inferiority of TWP compared to glucocorticoids, with results for total clinical remission (RR = 1.12, 95% CI: 0.84–1.50, I^2^ = 61.8%), 24hUP (WMD = −0.62, 95% CI: −1.96 to 0.72, I^2^ = 89.2%), and negative PLA2R reversion (RR = 1.31, 95% CI: 0.75–2.27, I^2^ = 77.1%).

Since Group 3 comprised only one study, no meta-analysis was conducted. After the 36-week treatment period, the study found similar outcomes between the TWP and tacrolimus groups regarding total remission, 24hUP, eGFR, and relapse (Liu et al., 2015).

### 3.4 Effectiveness of TWP on type 2 diabetic kidney disease

Four retrospective cohort studies and two RCTs evaluated the effectiveness of TWP in treating type 2 DKD (Ge et al., 2013; Wang, 2018; Xiong et al., 2020; Wang et al., 2022). All included patients were treated for an approximate period of 6 months. Based on the intervention strategies, the six studies were classified into two subgroups: Group 1 compared TWP plus routine medicines with routine medicines alone, and Group 2 compared TWP plus routine medicines with valsartan plus routine medicines.

**FIGURE 4:**
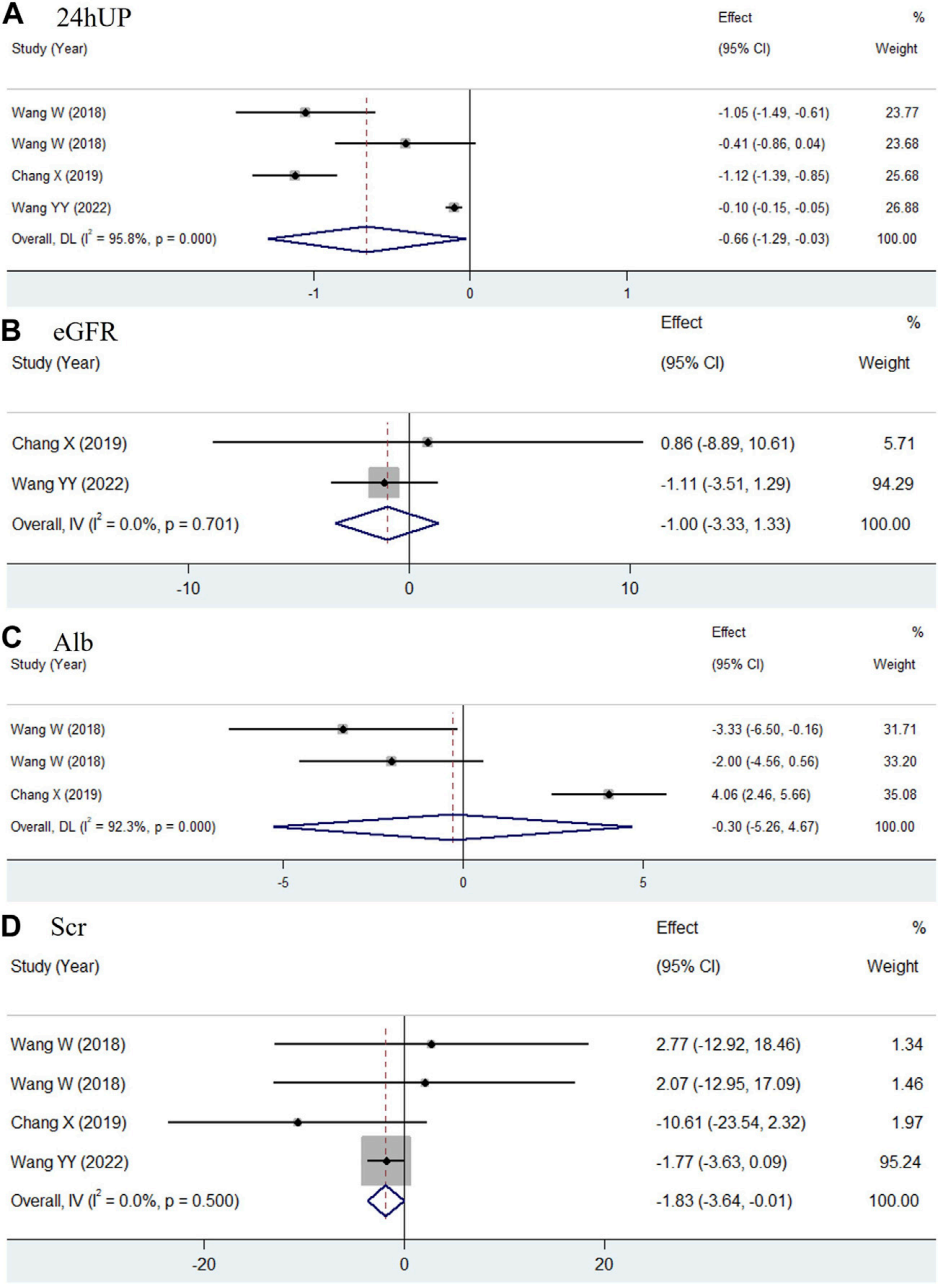
Forest plots comparing the efficacy of TWP + routine medicines with that of routine medicines in 24hUP **(A)**, eGFR **(B)**, Alb **(C)** and SCr **(D)** for type 2 DKD patients.

Four studies assessed the therapeutic outcomes of combining TWP with routine medicines, as compared to routine medicines alone (Wang, 2018; Xiong et al., 2020; Wang et al., 2022). The daily dose of TWP in these studies ranged from 30 to 60 mg. These studies were included in a meta-analysis, and the pooled outcomes for 24hUP, eGFR, Alb, and SCr after treatment are shown in Figure 4. The results indicated substantial heterogeneity in the pooled data: for 24hUP (WMD = −0.66, 95% CI: −1.29 to −0.03, I^2^ = 95.8%), for eGFR (WMD = −1.00, 95% CI: −3.33 to 1.33, I^2^ = 0.0%), for Alb (WMD = −0.3, 95% CI: −5.26 to 4.67, I^2^ = 92.3%), and for SCr (WMD = −1.83, 95% CI: −3.64 to −0.01, I^2^ = 0.0%). Given its ability to account for weight, sex, and age, eGFR is considered a more accurate measure of renal function than SCr. The addition of TWP was found to effectively reduce 24hUP levels without significantly impacting renal function or Alb levels.

**FIGURE 5:**
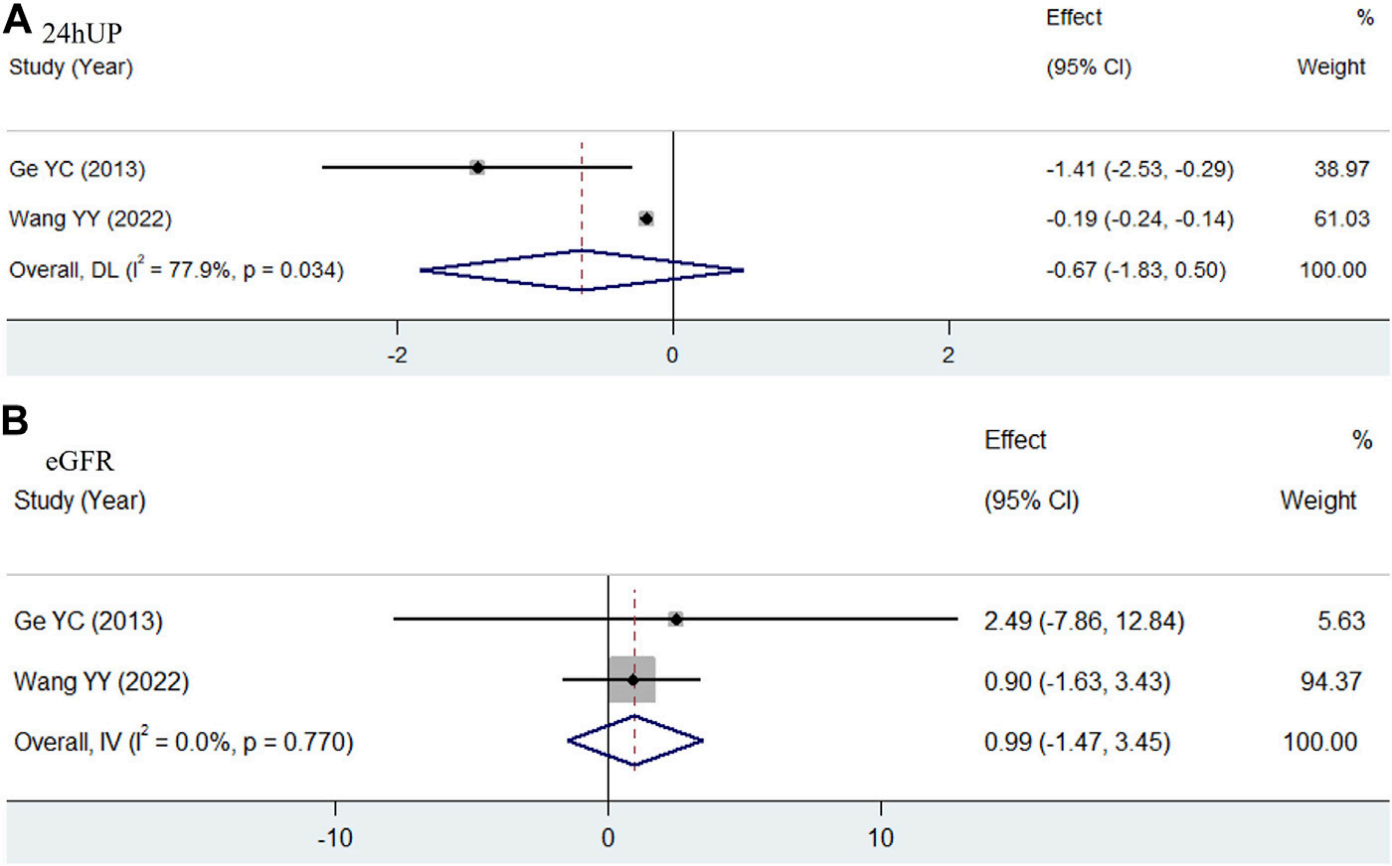
Meta-analysis forest plots of 24hUP **(A)** and eGFR **(B)** in type 2 DKD patients receiving TWP in comparison with that receiving valsartan.

Two studies evaluated the effectiveness of WP compared to valsartan in treating type 2 DKD (Ge et al., 2013; Wang et al., 2022). In both studies, the TWP groups received a uniform dose of 90 mg per day. As shown in Figure 5, the pooled results for 24hUP (WMD = −0.67, 95% CI: −1.83 to 0.50, I^2^ = 77.9%) and eGFR (WMD = 0.99, 95% CI: −1.47 to 3.45, I^2^ = 0.0%) indicated no significant differences between TWP and valsartan in terms of reducing urinary protein levels and improving renal function.

### 3.5 Effectiveness of TWP in Henoch-Schonlein purpura nephritis

Three retrospective cohorts, one prospective controlled open-label study, and one RCT investigated the effectiveness of adding TWP to conventional treatment in pediatric HSPN (Deng et al., 2010; Deng et al., 2012; Wu et al., 2013; Jin et al., 2021; Zhang et al., 2021). Clinical remission was reported in four of these five studies (Deng et al., 2010; Deng et al., 2012; Wu et al., 2013; Jin et al., 2021). However, the definition of clinical remission varied between studies, with ambiguity observed in one study (Jin et al., 2021) and differing units of measurement for proteinuria in another (Deng et al., 2010). Furthermore, the timing of reporting total clinical remission varied, one study reporting at 4 weeks (Deng et al., 2012) and another at 6 months (Wu et al., 2013). Due to these inconsistencies, clinical remission was not included in the meta-analysis.

**FIGURE 6:**
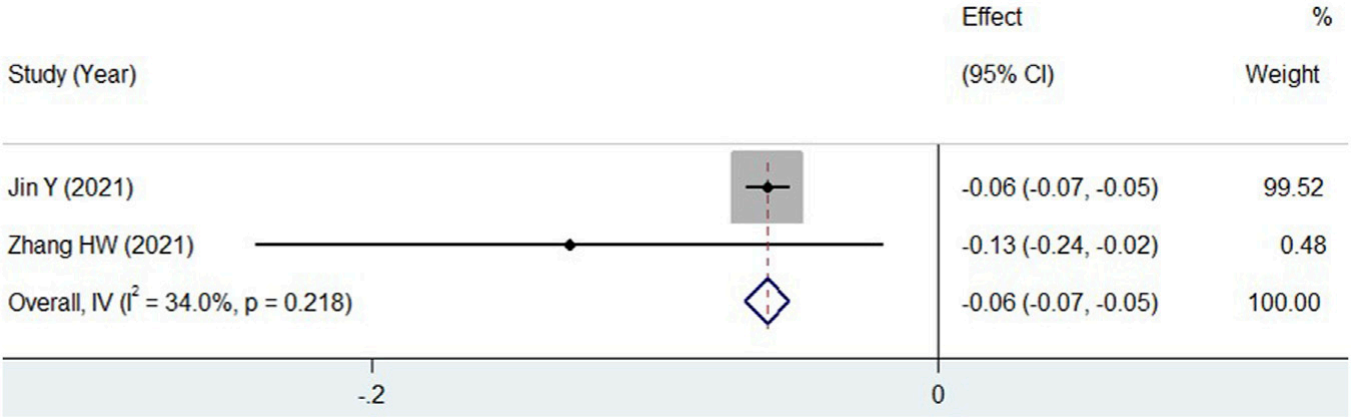
Meta-analysis forest plot of 24hUP in HSPN children with TWP addition to routine medicines.

For 24hUP, two studies provided data 12 weeks after treatment with additional TWP (Jin et al., 2021; Zhang et al., 2021). As shown in Figure 6, a significant decline in 24hUP was observed (WMD = −0.06, 95% CI: −0.07 to −0.05, I^2^ = 34.0%) in children treated with TWP plus routine medicines. Data for other indicators were not suitable for pooled analysis due to varying measurement units, low homogeneity, or because they were reported in only one study. The prospective use of TWP in treating pediatric HSPN is further discussed in the discussion section.

### 3.6 Safety of TWP on glomerulonephritis

ADRs were reported in all included studies. The recorded ADRs primarily included menstrual disorders, infections, hematologic toxicity, elevated transaminase levels, skin damage, and gastrointestinal discomfort. Since multiple ADRs could occur in the same patient, the aggregate count of ADR instances might exceed the number of affected patients. Therefore, the detailed incidence of each complication was individually compared. In the pooled analysis, leukopenia and anemia were classified as hematologic toxicity, pruritus and rash as skin damage, poor appetite, nausea, diarrhea, and vomiting as gastrointestinal discomfort.

**FIGURE 7:**
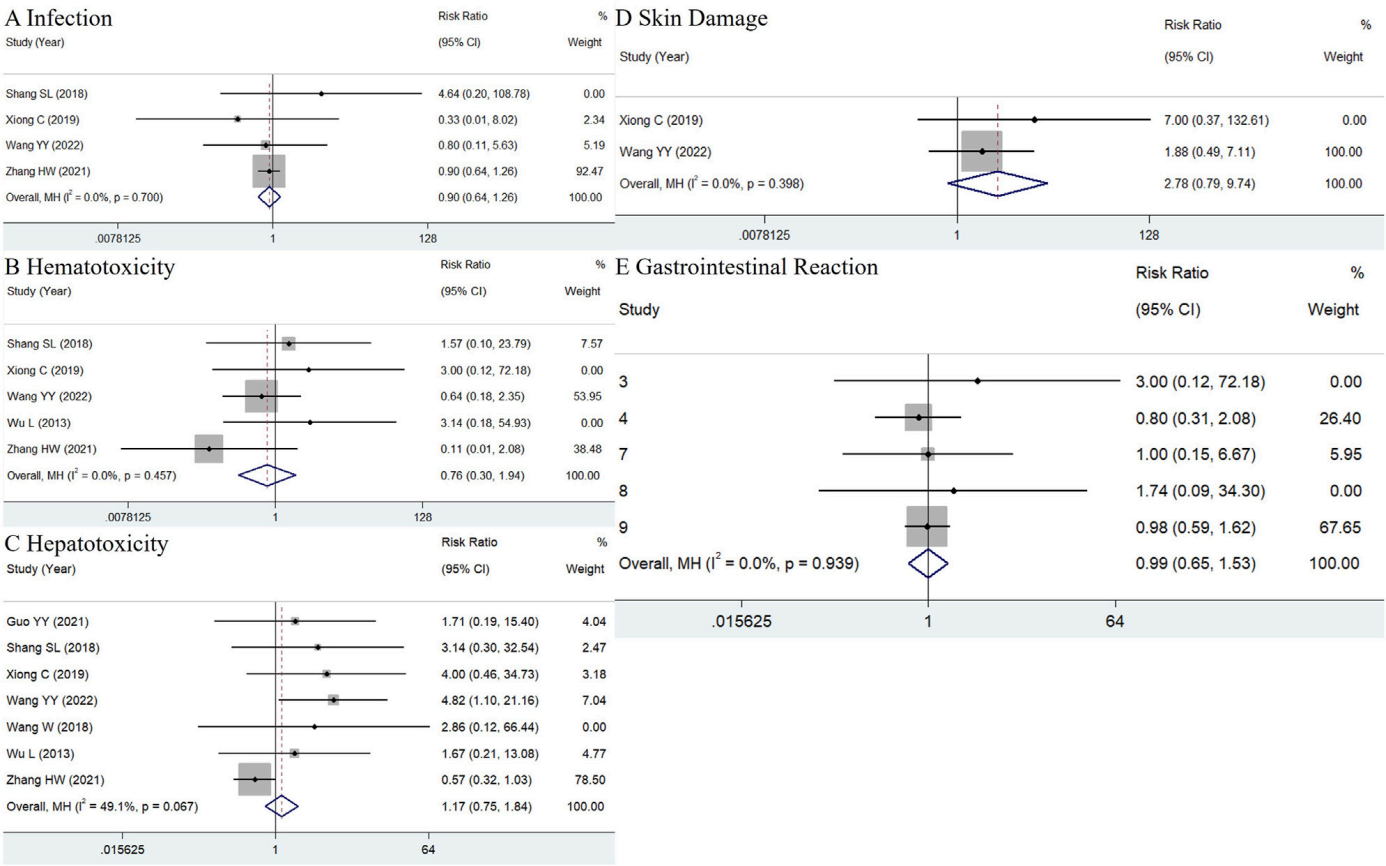
**(A)** Hematotoxicity **(B)**, hepatotoxicity **(C)**, skin damage **(D)** and gastrointestinal reaction **(E)** in TWP group just additionally treated with TWP besides the medicines used in control group.

Eleven studies were initially selected because they administered only TWP in addition to the control group’s medications. However, two of these studies were excluded because they reported only the total ADR incidence without describing specific ADRs. Consequently, nine studies were included in the meta-analysis (Wu et al., 2013; Shang et al., 2018; Wang, 2018; Guo et al., 2019; Xiong et al., 2020; Jin et al., 2021; Zhang et al., 2021; Wang et al., 2022). As shown in Figure 7, the pooled results did not indicate significant differences in the incidence of infections (RR = 0.9, 95% CI: 0.64–1.26, I^2^ = 0.0%), hematologic toxicity (RR = 0.76, 95% CI: 0.3–1.94, I^2^ = 0.0%), liver abnormalities (RR = 1.17, 95% CI: 0.75–1.84, I^2^ = 49.1%), skin damage (RR = 2.78, 95% CI: 0.79–9.74, I^2^ = 0.0%), and gastrointestinal discomfort (RR = 1.02, 95% CI: 0.66–1.58, I^2^ = 0.0%) between the TWP and control groups. However, the incidence of menstrual disturbances could not be quantitatively evaluated, as the number of women of childbearing age was not specified in all studies.

## 4 Discussion

Although it has a narrow therapeutic index, triptolide has been identified as the primary active component in TWP. Advances in extraction processes and refinery technology have allowed the removal of most harmful ingredients and the control of the triptolide content to less than 0.1%. Consequently, TWP exhibits both high effectiveness and low incidence of ADRs. Mechanisms by which triptolide mitigates renal damage likely include: (1) an immunosuppressive effect, which involves inhibiting antigen-driven proliferation of lymphocytes and promoting apoptosis of activated lymphocytes. This is accompanied by a reduction in serum antibody levels and the deposition of the membrane attack complex (MAC) in the glomerular capillaries (Zhao et al., 2018; Li et al., 2021; Dong et al., 2022); (2) anti-inflammatory and antifibrotic actions through a significant suppression of NF-kB signaling pathway activation and the overproduction of specific cytokines (Wan et al., 2020; Li et al., 2021; Dong et al., 2022); and (3) stabilization of the podocyte skeleton and maintenance of glomerular charge barrier integrity by directly regulating the expression of surface marker proteins (Ma et al., 2015; Wan et al., 2020).

### 4.1 Primary membranous nephropathy

With an increasing incidence, PMN is the second most common type of glomerulonephritis in China, accounting for 23.4% of cases (Xu et al., 2016), and is even the most prevalent in certain areas (Tang et al., 2017). The estimated prevalence of PMN in China has continued to increase, reaching 277 per million in 2021 (Thakur and Isherwood, 2021). Approximately two-thirds of patients with PMN are likely to progress to renal insufficiency. PMN is characterized by the sub-epithelial deposition of autoimmune complexes in the glomerular basement membrane. In 70%–80% of cases, these immune complexes are formed by the interaction of circulating autoantibodies with PLA2R, located *in situ* on the podocyte surface. These immune complexes can activate the complement system, leading to the generation of MAC and thus causing direct damage to GFB. The recruitment of MAC-induced inflammatory cells releases proteases, cytokines, and oxidants, further aggravating the injury to GFB and inducing fibrosis of the glomerular capillary wall.

Management of PMN focuses primarily on reducing proteinuria and modulating abnormal immune function. ARBs are preferentially recommended to decrease intrarenal pressure and proteinuria, provided the patient’s blood pressure can tolerate such treatment. For patients whose proteinuria is not adequately controlled with ARBs alone, a combination therapy of low-dose oral glucocorticoids and intravenous cyclophosphamide should be considered. If combination therapy is contraindicated, an oral calcineurin inhibitor may serve as an alternative. However, adverse events remain a significant concern, particularly for patients with pre-existing conditions.

Unlike other immunosuppressants, TWP offers protective effects in patients with PMN. The 24hUP level and the M-type PLA2R antibody are identified as high-risk factors for PMN. A reduction in proteinuria predicts a favorable prognosis. Given its high occurrence and near-100% specificity, detecting the PLA2R antibody is a serologically sensitive and practical method for monitoring therapeutic outcomes and assessing the prognosis of PMN. Integrating TWP with standard therapies has enhanced effects and reduced recurrence, maintaining good intergroup homogeneity. However, despite notable heterogeneity, pooled data from two other studies demonstrated comparability in total clinical remission, 24hUP, and negative PLA2R reversion between TWP and glucocorticoid treatments. For example, at enrollment, the 24hUP level in the TWP group (6.85 ± 1.79) was relatively higher than in the glucocorticoid group (6.16 ± 2.32) in the study by Gao et al. (2021), while in the study by Shang et al. (2018), the 24hUP level in the TWP group (6.61 ± 1.34) was relatively lower than in the glucocorticoid group (7.25 ± 1.61), leading to a more pronounced effect. Despite these differences, preliminary results suggest that TWP is a viable alternative to glucocorticoids, potentially avoiding steroid-related dependency and adverse drug reactions (ADRs). In a study by Liu SS, TWP’s therapeutic outcomes were shown to be non-inferior to those of tacrolimus, potentially reducing medical burdens due to its cost-effectiveness.

### 4.2 Diabetic kidney disease

In China, the number of individuals with diabetes has exceeded 114 million, representing approximately one-quarter of diabetic patients globally (Zhang et al., 2020). Of these, more than 90% have type 2 diabetes. DKD, a serious microvascular complication of diabetes, develops in approximately 40% of individuals with diabetes throughout their lifetime (Pan et al., 2022). Alarmingly, nearly 50% of those with DKD will progress to end-stage renal disease (ESRD) within their lifetime (Pan et al., 2022). Currently, DKD is one of the leading causes of mortality among diabetic patients. The pathogenesis of DKD is multifaceted. Long-term hyperglycemia is considered the primary catalyst that induces hemodynamic changes, disturbances in glucose metabolism, and the formation of advanced glycation end products (AGEs). These hemodynamic changes, increased blood flow, elevated arterial pressure, and hyperfiltration can damage GFB through shear stress, thus reducing GFR, activating RAS, and increasing intraglomerular pressure. This creates a detrimental cycle that exacerbates the decline in functioning nephrons. Furthermore, aberrant glucose metabolism and accumulation of AGEs contribute to the overproduction of reactive oxygen species and macrophage infiltration in the glomerular capillaries. This process promotes the release of pro-inflammatory and pro-fibrotic cytokines, ultimately leading to renal fibrosis and the cessation of filtration. These factors interact and synergistically advance the progression of DKD.

To slow the progression of DKD, current treatments focus on managing blood glucose levels, controlling blood pressure, and reducing proteinuria. Clinically, sodium-dependent glucose transporter 2 (SGLT2) inhibitors and ARBs are the primary options to mitigate proteinuria. SGLT2 inhibitors can protect the kidneys by promoting diuresis and natriuresis and enhancing glomeruli-tubular feedback. ARBs are effective in significantly inhibiting activated RAS and reducing intraglomerular pressure. Despite these benefits, the efficacy of both drugs has limitations. Additionally, the use of ARBs is controversial in patients with SCr levels >3 mg/dL or blood potassium levels >5.5 mmol/L, as described in the guidelines (Nephrology, 2021). SGLT2 inhibitors are not recommended for patients with a GFR below 30 mL/min/1.73 m^2^ (Association, 2021; Nephrology, 2021). Consequently, there is an urgent need to explore novel therapeutic options for DKD, particularly in cases with significant proteinuria.

Due to its anti-inflammatory and podocyte-protective properties, TWP may complement ARB and SGLT2 inhibitors, as the combination therapy targets several risk factors simultaneously (Huang et al., 2020). TWP potentially offers additional benefits in reducing urine protein levels, although its effects on enhancing eGFR and albumin levels are insignificant. Despite statistical heterogeneity, reduction in proteinuria suggests decreased glomerular damage and an improved prognosis. This heterogeneity probably arises from significantly lower baseline urine protein levels and a lower TWP dosage (30 mg per day) (Wang, 2018) compared to the other three studies (Wang, 2018; Xiong et al., 2020; Wang et al., 2022). Low-dose TWP often struggles to significantly reduce 24-h urine protein levels in patients with mild to moderate proteinuria (Ren et al., 2019). Moreover, no significant increase in eGFR was observed. Typically, a decrease in eGFR is only achieved with long-term treatment, and a 6-month period may not improve eGFR.

Regarding Alb levels, disparate results were observed in all studies. On the one hand, liver damage, a common adverse reaction to TWP, might interfere with Alb synthesis. On the other hand, TWP could increase Alb levels as a concurrent result of improved proteinuria. Consequently, the complex actions of TWP likely led to uncertain changes in Alb levels and significant heterogeneity between the studies. With marked heterogeneity between the two studies (Ge et al., 2013; Wang et al., 2022), the pooled results did not show statistical superiority of TWP in reducing proteinuria compared to valsartan. TWP was not superior to valsartan in reducing 24hUP levels at the end of treatment. However, it was superior in percentage reduction of urinary protein excretion. The minor superiority of TWP over valsartan could be attributed to normal baseline GFR levels (mean value above 90 mL/min/1.73 m^2^). In contrast, a significant decrease in 24hUP in the TWP group at the end of treatment end was perhaps linked to a low eGFR of <50 mL/min/1.73 m^2^, suggesting that therapeutic outcomes are more favorable in patients with impaired renal function. The preliminary results indicate that TWP could be an alternative option when ARB is contraindicated.

### 4.3 Henoch–Schonlein purpura nephritis

HSPN is a secondary impairment of the glomerular capillary network caused by IgA vasculitis (IgAV), predominantly affecting children. Approximately 90% of cases occur in children under 10 years of age. With an annual incidence of up to 20.4 per 100,000 children and 50% of IgAV cases involving HSPN, this condition represents about 13% of all childhood glomerular diseases (Gardner-Medwin et al., 2002; Chen and Mao, 2014; Jin et al., 2021). HSPN generally results from the deposition of immune complexes in the mesangium, subepithelial, or subendothelial areas, often involving circulating galactose-deficient IgA1 or antigens from group A streptococcal infections (Chen and Mao, 2014; Zhang et al., 2021). The successive activation of multiple steps leads to the overproduction of proinflammatory and profibrogenic mediators, ultimately damaging the glomerular filtration barrier.

Although most cases of HSPN in children show spontaneous recovery or have a favorable prognosis with standard medical treatment, approximately 20% may progress to CKD, and 1%–3% may advance to ESRD, particularly in severe cases characterized by nephrotic-range proteinuria, acute nephritis syndrome, or renal biopsy specimens showing cell crescents (Deng et al., 2012; Davin and Coppo, 2014). Furthermore, patients with severe renal involvement who receive suboptimal treatment are prone to progressive renal fibrosis (Deng et al., 2012). For example, patients with pathological grades II-III often experience worse outcomes than those with grades IV-V due to inadequate intervention (Ronkainen et al., 2002). In China, current treatment regimens for HSPN in children typically include ARBs, glucocorticoids, intravenous cyclophosphamide, and calcineurin inhibitors. Therefore, there is a critical need to identify new therapeutic agents to enhance treatment efficacy.

TWP has potential as an adjunctive treatment in managing HSPN, potentially leading to a favorable prognosis. Preliminary pooled results indicate that TWP enhances the anti-proteinuric effects when added to conventional therapy. Four studies evaluated the clinical outcomes of adding TWP to routine medications in HSPN children with nephrotic-range proteinuria (Deng et al., 2010; Deng et al., 2012; Wu et al., 2013). Adding TWP significantly increased the short-term remission rate compared to glucocorticoid alone (Wu et al., 2013). Long-term follow-up showed a lower incidence of active renal disease, renal insufficiency, or ESRD in patients receiving combined therapy (10%) compared to those on glucocorticoid monotherapy (28.6%) (Wu et al., 2013). Complete remission was achieved in all 24 children who received combined treatment by the fourth week, while only 2 out of 14 children managed with glucocorticoid alone reached remission; the addition of TWP helped the remaining 12 children achieve complete remission (Deng et al., 2010; Deng et al., 2012). The study by Zhang et al. compared the therapeutic outcomes of tacrolimus alone with a combination of TWP and tacrolimus in HSPN children with nephrotic-range proteinuria (Zhang et al., 2021). Although the initial levels of proteinuria and hematuria were similar under both treatment strategies, higher clinical control rates for these symptoms were observed with the combined therapy after a 2-year follow-up. Furthermore, adding TWP significantly reduced the recurrence induced by tacrolimus. Therefore, TWP can enhance the clinical remission rates of standard treatments in HSPN.

### 4.4 Safety of TWP

TWP-associated ADRs commonly included menstrual disorders, infections, hemocytopenia, elevated aminotransferases, skin damage, and gastrointestinal symptoms. The incidence of these ADRs varied considerably, likely influenced by factors such as research design, age of the participants, TWP dosage and duration, and concomitant medications. Reproductive toxicity is a significant concern, particularly for patients of childbearing age. None of the included studies reported sperm quality or count before and after treatment. Although cases of menstrual disturbance were documented, the total number of women of childbearing age was not specified, making it impossible to estimate the incidence of TWP-induced reproductive toxicity accurately. Animal experiments have demonstrated that TWP administration can damage testicles and sperm in male rats and uteruses in female rats (Jing et al., 2017; Guo et al., 2019). Reproductive toxicity is associated with disturbances in the hypothalamus-pituitary-gonadal axis and downregulation of key enzymes involved in the biosynthesis of sex hormones (Jing et al., 2017).

The meta-analysis revealed no statistically significant differences in infection, hematotoxicity, hepatotoxicity, skin damage, or gastrointestinal reactions. TWP does not appear to suppress the immune response excessively. Although TWP can substantially suppress activated peripheral blood lymphocytes, it has minimal effects on resting thymocytes (Yang et al., 1998). Among the ADRs, hepatotoxicity was the most common, with 41 cases reported in nine included studies. Liver damage associated with TWP is linked to downregulation of cytochrome P450 enzymes and P-glycoprotein, as well as increased oxidative stress, autophagy, hepatocyte apoptosis, cholestasis, and lipid accumulation (Hu et al., 2022). Hepatotoxicity, often presenting with leukopenia, is likely due to bone marrow suppression. Despite their high incidences, the mechanisms underlying gastrointestinal reactions and skin lesions were rarely reported.

The ADRs observed in the included studies were generally mild and well-tolerated, leading to temporary treatment discontinuation in only a few cases (Ge et al., 2013), with no patients withdrawing from treatment. According to the drug instructions, TWP should be administered at a dose of ≤1.5 mg/kg/day and for a treatment duration of ≤3 continuous months due to the dose-dependent nature of ADR incidence. As shown in Table 1, some subjects received TWP for longer than 3 months. It is speculated that ADRs would be more manageable if TWP were administered strictly according to drug guidelines. In clinical practice, specific medication reminders help patients quickly recognize ADRs and seek medical assistance. Additionally, hepatic function and routine blood tests should be monitored during treatment. In pediatric patients, no significant increase in ADRs was observed during the short-term follow-up of children with HSPN (Deng et al., 2010; Deng et al., 2012; Wu et al., 2013; Jin et al., 2021; Zhang et al., 2021). For adults with fertility concerns, sexual inhibition has not yet been investigated in controlled trials. Therefore, TWP should be prescribed only for these special populations in severe cases. Further exploration into the safety of TWP, particularly its potential anti-fertility effects, is needed with an extended observation period.

### 4.5 Strengths and limitations of this study

This meta-analysis presents several advantages and innovations. First, it includes all relevant studies published in English, expanding the scope of the investigation. Second, it is the first to comprehensively assess the therapeutic outcomes of TWP in both PMN and HSPN. Additionally, this analysis is the first to compare the effectiveness of TWP with valsartan in patients with DKD. The potential causes of heterogeneity were also analyzed, contributing to a deeper understanding of the treatment outcomes.

However, the study has certain limitations. First, the number of included studies and total cases was limited, reflecting the scarcity of published data. This paucity hindered the ability to perform subgroup analyses, sensitivity analyses, and assess publication bias. Second, considerable heterogeneity was observed among the included studies, likely due to variations in confounding factors such as baseline levels of 24hUP, blood pressure, and eGFR. Last, the unavailability of relevant data in three studies may have impacted the accuracy of the outcome assessments.

## 5 Conclusion

Adding TWP to existing treatment protocols for PMN, DKD, and severe HSPN in children can enhance effectiveness without introducing additional safety risks or substantial costs. Given its comparable efficacy, TWP is an alternative to glucocorticoids for PMN and valsartan for DKD, especially in cases where severe ADRs or contraindications preclude their use. Looking ahead, developing new formulations of *T. wilfordii* with reduced toxicity and enhanced effectiveness could broaden its clinical applications. However, the current results may be subject to bias, underlining the need for high-quality, prospective studies to further evaluate the efficacy and safety of TWP.

## Data Availability

The original contributions presented in the study are included in the article/Supplementary Material, further inquiries can be directed to the corresponding author.
