# Supplementary material for: Effectiveness and safety of tripterygium wilfordii poly-glycosides on glomerulonephritis: a systematic review and meta-analysis
Source: Front Pharmacol. 2024 May 22;15:1339153. doi: 10.3389/fphar.2024.1339153 (PMC11150713; doi:10.3389/fphar.2024.1339153)
Supplement: Supplementary file 1 [file Table1.docx]

Search strategies:

lupus nephritis：((((((((Lupus Glomerulonephritis) OR (Nephritis, Lupus)) OR (Lupus Nephritides)) OR (Nephritides, Lupus)) OR (Glomerulonephritis, Lupus)) OR (Glomerulonephritides, Lupus)) OR (Lupus Glomerulonephritides)) OR (Lupus Nephritis)) AND ((((((((((((((((((((Tripterygiums) OR (Tripterygium wilfordii)) OR (Tripterygium wilfordius)) OR (wilfordius, Tripterygium)) OR (Leigong Teng)) OR (Leigong Tengs)) OR (Teng, Leigong)) OR (Tengs, Leigong)) OR (Thundergod Vine)) OR (Thundergod Vines)) OR (Vine, Thundergod)) OR (Vines, Thundergod)) OR (Tripterygium hypoglaucum)) OR (Tripterygium hypoglaucums)) OR (hypoglaucums, Tripterygium)) OR (Tripterygium)) OR (triptolide)) OR (Triptolidenol)) OR (Glucosidorum Tripterygll Totorum)) OR (Tripterysium Glycosides))

Focal Segmental Glomerulosclerosis: ((((((((((((((((Segmental Glomerulosclerosis, Focal) OR (Glomerulosclerosis, Focal)) OR (Focal Glomerulosclerosis)) OR (Focal Segmental Glomerulosclerosis)) OR (Glomerulonephritis, Focal Sclerosing)) OR (Focal Sclerosing Glomerulonephritides)) OR (Focal Sclerosing Glomerulonephritis)) OR (Glomerulonephritides, Focal Sclerosing)) OR (Sclerosing Glomerulonephritides, Focal)) OR (Sclerosing Glomerulonephritis, Focal)) OR (Hyalinosis, Segmental Glomerular)) OR (Glomerular Hyalinosis, Segmental)) OR (Segmental Glomerular Hyalinosis)) OR (Hyalinosis, Segmental)) OR (Segmental Hyalinosis)) OR (Glomerulosclerosis, Focal Segmental)) AND ((((((((((((((((((((Tripterygiums) OR (Tripterygium wilfordii)) OR (Tripterygium wilfordius)) OR (wilfordius, Tripterygium)) OR (Leigong Teng)) OR (Leigong Tengs)) OR (Teng, Leigong)) OR (Tengs, Leigong)) OR (Thundergod Vine)) OR (Thundergod Vines)) OR (Vine, Thundergod)) OR (Vines, Thundergod)) OR (Tripterygium hypoglaucum)) OR (Tripterygium hypoglaucums)) OR (hypoglaucums, Tripterygium)) OR (Tripterygium)) OR (triptolide)) OR (Triptolidenol)) OR (Glucosidorum Tripterygll Totorum)) OR (Tripterysium Glycosides))

IgA Glomerulonephritides：((((((((((((((Glomerulonephritides, IGA) OR (Berger's Disease)) OR (Bergers Disease)) OR (IGA Glomerulonephritis)) OR (Nephropathy, IGA)) OR (Iga Nephropathy 1)) OR (Nephropathy 1, Iga)) OR (Immunoglobulin A Nephropathy)) OR (Nephropathy, Immunoglobulin A)) OR (Nephritis, IGA Type)) OR (IGA Type Nephritis)) OR (Berger Disease)) OR (IGA Nephropathy)) OR (Glomerulonephritis, IGA)) AND ((((((((((((((((((((Tripterygiums) OR (Tripterygium wilfordii)) OR (Tripterygium wilfordius)) OR (wilfordius, Tripterygium)) OR (Leigong Teng)) OR (Leigong Tengs)) OR (Teng, Leigong)) OR (Tengs, Leigong)) OR (Thundergod Vine)) OR (Thundergod Vines)) OR (Vine, Thundergod)) OR (Vines, Thundergod)) OR (Tripterygium hypoglaucum)) OR (Tripterygium hypoglaucums)) OR (hypoglaucums, Tripterygium)) OR (Tripterygium)) OR (triptolide)) OR (Triptolidenol)) OR (Glucosidorum Tripterygll Totorum)) OR (Tripterysium Glycosides))

Anti Glomerular Basement Membrane Disease：((((((((((Anti Glomerular Basement Membrane Disease) OR (Anti-GBM Disease)) OR (Anti GBM Disease)) OR (Lung Purpura with Nephritis)) OR (Goodpasture's Syndrome)) OR (Goodpastures Syndrome)) OR (Syndrome, Goodpasture's)) OR (Goodpasture Syndrome)) OR (Syndrome, Goodpasture)) OR (Anti-Glomerular Basement Membrane Disease)) AND ((((((((((((((((((((Tripterygiums) OR (Tripterygium wilfordii)) OR (Tripterygium wilfordius)) OR (wilfordius, Tripterygium)) OR (Leigong Teng)) OR (Leigong Tengs)) OR (Teng, Leigong)) OR (Tengs, Leigong)) OR (Thundergod Vine)) OR (Thundergod Vines)) OR (Vine, Thundergod)) OR (Vines, Thundergod)) OR (Tripterygium hypoglaucum)) OR (Tripterygium hypoglaucums)) OR (hypoglaucums, Tripterygium)) OR (Tripterygium)) OR (triptolide)) OR (Triptolidenol)) OR (Glucosidorum Tripterygll Totorum)) OR (Tripterysium Glycosides))

Membranous Glomerulonephritides ：(((((((((((((((((((((((Membranous Glomerulonephritides) OR (Glomerulonephritides, Membranous)) OR (Membranous Glomerulonephritis)) OR (Nephropathy, Membranous)) OR (Membranous Glomerulopathy)) OR (Glomerulopathy, Membranous)) OR (Membranous Nephropathy)) OR (Extramembranous Glomerulopathy)) OR (Glomerulopathy, Extramembranous)) OR (Membranous Glomerulonephropathy)) OR (Glomerulonephropathy, Membranous)) OR (Heymann Nephritis)) OR (Nephritis, Heymann)) OR (Idiopathic Membranous Glomerulonephritis)) OR (Glomerulonephritides, Idiopathic Membranous)) OR (Glomerulonephritis, Idiopathic Membranous)) OR (Idiopathic Membranous Glomerulonephritides)) OR (Membranous Glomerulonephritides, Idiopathic)) OR (Membranous Glomerulonephritis, Idiopathic)) OR (Idiopathic Membranous Nephropathy)) OR (Membranous Nephropathy, Idiopathic)) OR (Nephropathy, Idiopathic Membranous)) OR (Glomerulonephritis, Membranous)) AND ((((((((((((((((((((Tripterygiums) OR (Tripterygium wilfordii)) OR (Tripterygium wilfordius)) OR (wilfordius, Tripterygium)) OR (Leigong Teng)) OR (Leigong Tengs)) OR (Teng, Leigong)) OR (Tengs, Leigong)) OR (Thundergod Vine)) OR (Thundergod Vines)) OR (Vine, Thundergod)) OR (Vines, Thundergod)) OR (Tripterygium hypoglaucum)) OR (Tripterygium hypoglaucums)) OR (hypoglaucums, Tripterygium)) OR (Tripterygium)) OR (triptolide)) OR (Triptolidenol)) OR (Glucosidorum Tripterygll Totorum)) OR (Tripterysium Glycosides))

Membranoproliferative Glomerulonephritides：((((((((((((((((((((((((((((((((((((((Glomerulonephritides, Membranoproliferative) OR (Membranoproliferative Glomerulonephritides)) OR (Membranoproliferative Glomerulonephritis)) OR (Glomerulonephritis, Hypocomplementemic)) OR (Hypocomplementemic Glomerulonephritides)) OR (Hypocomplementemic Glomerulonephritis)) OR (MPGN Membranoproliferative Glomerulonephritis)) OR (Glomerulonephritides, MPGN Membranoproliferative)) OR (Glomerulonephritis, MPGN Membranoproliferative)) OR (MPGN Membranoproliferative Glomerulonephritides)) OR (Membranoproliferative Glomerulonephritides, MPGN)) OR (Membranoproliferative Glomerulonephritis, MPGN)) OR (Glomerulonephritis, Mesangiocapillary)) OR (Mesangiocapillary Glomerulonephritides)) OR (Mesangiocapillary Glomerulonephritis)) OR (Membranoproliferative Glomerulonephritis, Type I)) OR (Subendothelial Membranoproliferative Glomerulonephritis)) OR (Membranoproliferative Glomerulonephritis, Subendothelial)) OR (Mesangiocapillary Glomerulonephritis, Type I)) OR (Complement 3 Glomerulopathies)) OR (Glomerulopathies, Complement 3)) OR (Glomerulopathy, Complement 3)) OR (C3G Complement 3 Glomerulopathy)) OR (Complement 3 Glomerulopathy)) OR (Membranoproliferative Glomerulonephritis, Type III)) OR (Membranoproliferative Glomerulonephritis, Type II)) OR (Dense Deposit Disease)) OR (Type II MPGN)) OR (MPGN, Type II)) OR (Type II MPGNs)) OR (Mesangiocapillary Glomerulonephritis, Type II)) OR (MPGNII)) OR (MPGNIIs)) OR (DDD MPGNII)) OR (DDD MPGNIIs)) OR (MPGNII, DDD)) OR (Membranoproliferative Glomerulonephritis Type II)) OR (Glomerulonephritis, Membranoproliferative)) AND ((((((((((((((((((((Tripterygiums) OR (Tripterygium wilfordii)) OR (Tripterygium wilfordius)) OR (wilfordius, Tripterygium)) OR (Leigong Teng)) OR (Leigong Tengs)) OR (Teng, Leigong)) OR (Tengs, Leigong)) OR (Thundergod Vine)) OR (Thundergod Vines)) OR (Vine, Thundergod)) OR (Vines, Thundergod)) OR (Tripterygium hypoglaucum)) OR (Tripterygium hypoglaucums)) OR (hypoglaucums, Tripterygium)) OR (Tripterygium)) OR (triptolide)) OR (Triptolidenol)) OR (Glucosidorum Tripterygll Totorum)) OR (Tripterysium Glycosides))

Diabetic Nephropathies：((((((((((((((((((Nephropathies, Diabetic) OR (Diabetic Nephropathies)) OR (Nephropathy, Diabetic)) OR (Diabetic Nephropathy)) OR (Diabetic Kidney Disease)) OR (Diabetic Kidney Diseases)) OR (Kidney Disease, Diabetic)) OR (Kidney Diseases, Diabetic)) OR (Diabetic Glomerulosclerosis)) OR (Glomerulosclerosis, Diabetic)) OR (Intracapillary Glomerulosclerosis)) OR (Nodular Glomerulosclerosis)) OR (Glomerulosclerosis, Nodular)) OR (Kimmelstiel-Wilson Syndrome)) OR (Kimmelstiel Wilson Syndrome)) OR (Syndrome, Kimmelstiel-Wilson)) OR (Kimmelstiel-Wilson Disease)) OR (Kimmelstiel Wilson Disease)) AND ((((((((((((((((((((Tripterygiums) OR (Tripterygium wilfordii)) OR (Tripterygium wilfordius)) OR (wilfordius, Tripterygium)) OR (Leigong Teng)) OR (Leigong Tengs)) OR (Teng, Leigong)) OR (Tengs, Leigong)) OR (Thundergod Vine)) OR (Thundergod Vines)) OR (Vine, Thundergod)) OR (Vines, Thundergod)) OR (Tripterygium hypoglaucum)) OR (Tripterygium hypoglaucums)) OR (hypoglaucums, Tripterygium)) OR (Tripterygium)) OR (triptolide)) OR (Triptolidenol)) OR (Glucosidorum Tripterygll Totorum)) OR (Tripterysium Glycosides))

Chronic Renal Insufficiencies：(((((((((((((((((((Chronic Renal Insufficiencies) OR (Renal Insufficiencies, Chronic)) OR (Chronic Renal Insufficiency)) OR (Kidney Insufficiency, Chronic)) OR (Chronic Kidney Insufficiency)) OR (Chronic Kidney Insufficiencies)) OR (Kidney Insufficiencies, Chronic)) OR (Chronic Kidney Diseases)) OR (Chronic Kidney Disease)) OR (Disease, Chronic Kidney)) OR (Diseases, Chronic Kidney)) OR (Kidney Disease, Chronic)) OR (Kidney Diseases, Chronic)) OR (Chronic Renal Diseases)) OR (Chronic Renal Disease)) OR (Disease, Chronic Renal)) OR (Diseases, Chronic Renal)) OR (Renal Disease, Chronic)) OR (Renal Diseases, Chronic)）AND ((((((((((((((((((((Tripterygiums) OR (Tripterygium wilfordii)) OR (Tripterygium wilfordius)) OR (wilfordius, Tripterygium)) OR (Leigong Teng)) OR (Leigong Tengs)) OR (Teng, Leigong)) OR (Tengs, Leigong)) OR (Thundergod Vine)) OR (Thundergod Vines)) OR (Vine, Thundergod)) OR (Vines, Thundergod)) OR (Tripterygium hypoglaucum)) OR (Tripterygium hypoglaucums)) OR (hypoglaucums, Tripterygium)) OR (Tripterygium)) OR (triptolide)) OR (Triptolidenol)) OR (Glucosidorum Tripterygll Totorum)) OR (Tripterysium Glycosides))

Rapidly progressive glomerulonephritis： (((((((Rapidly progressive glomerulonephritis) OR (Glomerulonephritis - pulmonary hemorrhage)) OR (Pulmonary renal syndrome)) OR (Anti-glomerular basement membrane antibody disease)) OR (Crescentic Glomerulonephritis))) OR (extracapillary proliferative glomerulonephritis)) AND ((((((((((((((((((((Tripterygiums) OR (Tripterygium wilfordii)) OR (Tripterygium wilfordius)) OR (wilfordius, Tripterygium)) OR (Leigong Teng)) OR (Leigong Tengs)) OR (Teng, Leigong)) OR (Tengs, Leigong)) OR (Thundergod Vine)) OR (Thundergod Vines)) OR (Vine, Thundergod)) OR (Vines, Thundergod)) OR (Tripterygium hypoglaucum)) OR (Tripterygium hypoglaucums)) OR (hypoglaucums, Tripterygium)) OR (Tripterygium)) OR (triptolide)) OR (Triptolidenol)) OR (Glucosidorum Tripterygll Totorum)) OR (Tripterysium Glycosides))

Glomerulonephritis： (((((Glomerulonephritis) OR (Glomerulonephritides)) OR (Kidney Scarring)) OR (Scarring, Kidney)) OR (Bright Disease)) AND ((((((((((((((((((((Tripterygiums) OR (Tripterygium wilfordii)) OR (Tripterygium wilfordius)) OR (wilfordius, Tripterygium)) OR (Leigong Teng)) OR (Leigong Tengs)) OR (Teng, Leigong)) OR (Tengs, Leigong)) OR (Thundergod Vine)) OR (Thundergod Vines)) OR (Vine, Thundergod)) OR (Vines, Thundergod)) OR (Tripterygium hypoglaucum)) OR (Tripterygium hypoglaucums)) OR (hypoglaucums, Tripterygium)) OR (Tripterygium)) OR (triptolide)) OR (Triptolidenol)) OR (Glucosidorum Tripterygll Totorum)) OR (Tripterysium Glycosides))

Nephrotic Syndrome： (((((((((((((((((((((Nephrotic Syndrome) OR (Nephrotic Syndromes)) OR (Syndrome, Nephrotic)) OR (Steroid-Dependent Nephrotic Syndrome)) OR (Nephrotic Syndrome, Steroid-Dependent)) OR (Steroid Dependent Nephrotic Syndrome)) OR (Steroid-Dependent Nephrotic Syndromes)) OR (Childhood Idiopathic Nephrotic Syndrome)) OR (Pediatric Idiopathic Nephrotic Syndrome)) OR (Multi-Drug Resistant Nephrotic Syndrome)) OR (Multi Drug Resistant Nephrotic Syndrome)) OR (Steroid-Sensitive Nephrotic Syndrome)) OR (Nephrotic Syndrome, Steroid-Sensitive)) OR (Steroid Sensitive Nephrotic Syndrome)) OR (Steroid-Sensitive Nephrotic Syndromes)) OR (Syndrome, Steroid-Sensitive Nephrotic)) OR (Steroid-Resistant Nephrotic Syndrome)) OR (Nephrotic Syndrome, Steroid-Resistant)) OR (Steroid Resistant Nephrotic Syndrome)) OR (Steroid-Resistant Nephrotic Syndromes)) OR (Frequently Relapsing Nephrotic Syndrome)) AND ((((((((((((((((((((Tripterygiums) OR (Tripterygium wilfordii)) OR (Tripterygium wilfordius)) OR (wilfordius, Tripterygium)) OR (Leigong Teng)) OR (Leigong Tengs)) OR (Teng, Leigong)) OR (Tengs, Leigong)) OR (Thundergod Vine)) OR (Thundergod Vines)) OR (Vine, Thundergod)) OR (Vines, Thundergod)) OR (Tripterygium hypoglaucum)) OR (Tripterygium hypoglaucums)) OR (hypoglaucums, Tripterygium)) OR (Tripterygium)) OR (triptolide)) OR (Triptolidenol)) OR (Glucosidorum Tripterygll Totorum)) OR (Tripterysium Glycosides))

purpura Glomerulonephritis：((((((((purpura Glomerulonephritis) OR (purpura Nephritis)) OR (purpura Nephritides)) OR (purpura Glomerulonephritides)) OR (purpura Nephropathy)) OR (purpura Glomerulopathy)) OR (purpura Glomerulonephropathy)) OR (HSPN)) AND ((((((((((((((((((((Tripterygiums) OR (Tripterygium wilfordii)) OR (Tripterygium wilfordius)) OR (wilfordius, Tripterygium)) OR (Leigong Teng)) OR (Leigong Tengs)) OR (Teng, Leigong)) OR (Tengs, Leigong)) OR (Thundergod Vine)) OR (Thundergod Vines)) OR (Vine, Thundergod)) OR (Vines, Thundergod)) OR (Tripterygium hypoglaucum)) OR (Tripterygium hypoglaucums)) OR (hypoglaucums, Tripterygium)) OR (Tripterygium)) OR (triptolide)) OR (Triptolidenol)) OR (Glucosidorum Tripterygll Totorum)) OR (Tripterysium Glycosides))

Lipoid Nephroses： (((((((((((((((((((((((Nephrosis, Lipoid) OR (Lipoid Nephroses)) OR (Lipoid Nephrosis)) OR (Nephroses, Lipoid)) OR (Minimal Change Glomerulopathy)) OR (Minimal Change Disease)) OR (Change Diseases, Minimal)) OR (Disease, Minimal Change)) OR (Diseases, Minimal Change)) OR (Minimal Change Diseases)) OR (Nephropathy, Minimal Change)) OR (Minimal Change Nephropathies)) OR (Minimal Change Nephropathy)) OR (Nephropathies, Minimal Change)) OR (Glomerulopathy, Minimal Change)) OR (Glomerulopathies, Minimal Change)) OR (Idiopathic Minimal Change Nephrotic Syndrome)) OR (Nephrotic Syndrome, Minimal Change)) OR (Minimal Change Nephrotic Syndrome)) OR (Glomerulonephritis, Minimal Change)) OR (Glomerulonephritides, Minimal Change)) OR (Minimal Change Glomerulonephritides)) OR (Minimal Change Glomerulonephritis)) AND ((((((((((((((((((((Tripterygiums) OR (Tripterygium wilfordii)) OR (Tripterygium wilfordius)) OR (wilfordius, Tripterygium)) OR (Leigong Teng)) OR (Leigong Tengs)) OR (Teng, Leigong)) OR (Tengs, Leigong)) OR (Thundergod Vine)) OR (Thundergod Vines)) OR (Vine, Thundergod)) OR (Vines, Thundergod)) OR (Tripterygium hypoglaucum)) OR (Tripterygium hypoglaucums)) OR (hypoglaucums, Tripterygium)) OR (Tripterygium)) OR (triptolide)) OR (Triptolidenol)) OR (Glucosidorum Tripterygll Totorum)) OR (Tripterysium Glycosides))

Endocapillary proliferative glomerulonephritis：((((((Endocapillary proliferative glomerulonephritis) OR (diffuse proliferative glomerulonephritis)) OR (acute glomerulonephritis)) OR (Endocapillary proliferative nephritis)) OR (diffuse proliferative nephritis)) OR (acute nephritis)) AND ((((((((((((((((((((Tripterygiums) OR (Tripterygium wilfordii)) OR (Tripterygium wilfordius)) OR (wilfordius, Tripterygium)) OR (Leigong Teng)) OR (Leigong Tengs)) OR (Teng, Leigong)) OR (Tengs, Leigong)) OR (Thundergod Vine)) OR (Thundergod Vines)) OR (Vine, Thundergod)) OR (Vines, Thundergod)) OR (Tripterygium hypoglaucum)) OR (Tripterygium hypoglaucums)) OR (hypoglaucums, Tripterygium)) OR (Tripterygium)) OR (triptolide)) OR (Triptolidenol)) OR (Glucosidorum Tripterygll Totorum)) OR (Tripterysium Glycosides))

Table S1. The particular points of quality score for cohort studies and prospective controlled open-label study

| Study | Selection | | | | Comparability | Outcome | | |
| --- | --- | --- | --- | --- | --- | --- | --- | --- |
|  | Representativeness of intervention cohort | Selection of non-inervention cohort | Ascertainment of intervention | Outcome not present at start of study |  | Outcome assessment | Long enough follow-up | Adequacy of follow up |
| Shanshan Liu | ※ | ※ |  | ※ | ※ | ※ | ※ | ※ |
| ShunLai Shang | ※ | ※ |  | ※ | ※※ | ※ | ※ | ※ |
| ShunLai Shang | ※ | ※ |  | ※ | ※※ | ※ | ※ | ※ |
| Ying Gao | ※ | ※ |  | ※ | ※ | ※ | ※ | ※ |
| Yuanyuan Guo | ※ | ※ |  | ※ | ※※ | ※ | ※ | ※ |
| YouYun Wang | ※ | ※ |  | ※ | ※※ | ※ | ※ | ※ |
| YouYun Wang | ※ | ※ |  | ※ | ※※ | ※ | ※ | ※ |
| Fang Deng | ※ | ※ |  | ※ |  | ※ | ※ | ※ |
| Fang Deng | ※ | ※ |  | ※ | ※ | ※ | ※ | ※ |
| Li Wu | ※ | ※ |  | ※ | ※ | ※ | ※ | ※ |
| Yan Jin | ※ | ※ |  | ※ |  | ※ | ※ | ※ |

Table S2. The particular points of quality score for RCTs

| Study | Randomization | Proper randomization method | Blinding | Proper blinding method | Withdrawals and dropouts | Inclusion/exclusion criteria | Adverse effects | Statistical analysis |
| --- | --- | --- | --- | --- | --- | --- | --- | --- |
| Yongchun Ge | 1 | 0 | 0 | 0 | 1 | 1 | 1 | 1 |
| Wei Wang | 1 | 1 | 0 | 0 | 1 | 1 | 1 | 1 |
| Wei Wang | 1 | 1 | 0 | 0 | 1 | 1 | 1 | 1 |
| Chang Xiong | 1 | 1 | 0 | 0 | 1 | 1 | 1 | 1 |
| Huiwu Zhang | 1 | 1 | 0 | 0 | 1 | 1 | 1 | 1 |
